# The relationship between psychological distress and cognitive failure among breast cancer survivors: a network analysis

**DOI:** 10.3389/fpsyg.2024.1420125

**Published:** 2024-07-10

**Authors:** Bingxue Han, Jialin Yan, Ruoyu Xiong, Miaomiao Wang, Jinxia Liu, Liping Jia, Jinhua Dou, Xiaoli Liu, Huaju Fan, Jianying Li, Caiyun Zhang, Xiuhong Sun, He Du, Yufeng Ma, Shuai Teng, Nengzhi Jiang, Guohua Lu

**Affiliations:** ^1^School of Public Health, Shandong Second Medical University, Weifang, Shandong, China; ^2^School of Psychology, Shandong Second Medical University, Weifang, Shandong, China; ^3^College of Teacher Education, Weifang University, Weifang, Shandong, China; ^4^Department of Thyroid and Breast Surgery, Weifang Hospital of Traditional Chinese Medicine, Weifang, Shandong, China; ^5^Student Affairs Department, Shandong Second Medical University, Weifang, Shandong, China; ^6^Department of Thyroid and Breast Surgery, Affiliated Hospital of Shandong Second Medical University, Weifang, Shandong, China; ^7^Psychological Counseling Center, Weifang University, Weifang, Shandong, China

**Keywords:** breast cancer, psychological distress, cognitive failure, network analysis, depression

## Abstract

**Background:**

Psychological distress is highly prevalent and has a severe impact on the quality of life among breast cancer survivors. This type of distress is associated with cognitive failure. However, previous studies have focused solely on the total scale scores of these two concepts while ignoring the unique relationship between specific components. In the present study, we utilized network analysis to explore the relationship between psychological distress and cognitive failure in breast cancer survivors.

**Methods:**

The network analysis approach was adopted to estimate the regularized partial correlation network in a cross-sectional sample of 409 breast cancer survivors. All participants were assessed using the Depression Anxiety Stress Scale and the Cognitive Failure Questionnaire. The Gaussian Graphical Model was employed to estimate the network, centrality indices, and edge weights, providing a description of the characteristics of the network.

**Results:**

The results indicated that anxiety–stress and depression–stress were the strongest edges in the community of psychological distress. Distractibility–memory was the strongest edge in the community of cognitive failure. Distractibility and memory were the most central nodes, with the highest expected influence in the network. Depression and motor coordination acted as important bridge nodes with the highest bridge expected influence.

**Conclusion:**

Distractibility and memory in cognitive failure played important roles in activating and maintaining the relationship network. Motor coordination was identified as the crucial pathway for the impact of cognitive failure on psychological distress. Interventions targeting these specific issues might be more effective in improving cognitive failure and reducing psychological distress among breast cancer survivors.

## 1 Introduction

Breast cancer is the most prevalent cancer and the main cause of cancer death among women worldwide (Sung et al., 2021). It is also the most rapidly growing cancer in China (Sun et al., 2023). The diagnosis and treatment of breast cancer, both acting as severe stressors, can result in significant cognitive impairments and emotional problems for patients (Wirkner et al., 2017). In particular, anxiety and depression are more prevalent in breast cancer survivors. In previous research, 32% of breast cancer survivors reported depression (Pilevarzadeh et al., 2019), and 41.9% suffered from anxiety (Hashemi et al., 2020). Psychological distress, including anxiety and depression, can occur throughout treatment and persist during breast cancer rehabilitation (Xu et al., 2022). Anxiety and depression are correlated with poor physical functioning and an increased mortality risk (Wang et al., 2020) and may lead to more physical examinations and excessive use of medications (Otto et al., 2018). Hence, addressing the psychological distress experienced by breast cancer survivors is important.

Researchers have suggested that anxiety and depression arise as a result of individual failures in attention, memory, action, and interpersonal relationships (Zinchenko et al., 2015; Fisher et al., 2020). Cognitive failure refers to frequent lapses that disrupt normal actions (physical or mental), acting as a barrier to cognitive control (Carrigan and Barkus, 2016). According to information processing theory, an individual's cognitive resources are limited (Franconeri et al., 2013). More cognitive resources are needed to process complex stimuli. The diagnosis and treatment of breast cancer demand the use of more cognitive resources, and breast cancer survivors are more likely to experience cognitive failure and psychological distress. For instance, a systematic review of subjective cognitive dysfunction in breast cancer survivors confirmed that they experienced cognitive failure, such as memory and concentration problems, in their daily lives. Importantly, these cognitive failures were indicative of anxiety, depression, and general psychological distress (Pullens et al., 2010). Previous studies have found a relationship between cognitive failure and psychological distress in bereaved individuals (Fisher et al., 2020) and intensive care unit survivors (Brück et al., 2019). Self-reported cognitive failure is an indicative factor of a general ruminative cognitive style, which increases vulnerability to mental health problems, including negative affect (Bridger et al., 2011; Payne and Schnapp, 2014). However, previous research has focused solely on the total scores when studying the relationship between cognitive failure and psychological distress. The correlations between different components of cognitive failure and psychological distress in breast cancer survivors have not been well elucidated. Moreover, the key components of cognitive failure that impact psychological distress have not been clearly identified. Clarifying these components would be beneficial in helping breast cancer survivors improve their cognitive abilities, reduce psychological distress, and enhance their quality of life.

The network analysis provides a new perspective when clarifying the relationship between variables, which have been chiefly explored from the perspective of latent variables. Network theory is increasingly being adopted as a novel approach to understanding psychopathological nature and treatment (Rogers et al., 2019). Network analysis postulates that different psychological structures interact, and changes in one structure may trigger alterations in another. This process also occurs for diverse symptom expressions within the same psychological structure (Borsboom, 2008). A network structure is composed of nodes and edges. Each node represents a distinct psychological symptom or trait, while edges connect the nodes. Network analysis identifies central nodes as the crucial symptoms or traits of psychological structures. Central nodes activate entire networks; play a significant role in disease generation, maintenance, and progression; and are the direct and key targets for prevention and therapy (Fried et al., 2017).

Furthermore, network analysis visualizes the connectivity between different psychological structures. Bridge nodes are considered “bridges” among various psychological traits. For example, a recent network analysis revealed that the fear of cancer recurrence, anxiety, and depression in breast cancer survivors were connected by the important bridge nodes of “feeling afraid” (a symptom of anxiety), “uncontrollable worry” (a symptom of anxiety), “restlessness” (a symptom of anxiety), and “moving slowly or being restless” (a symptom of depression) (Yang et al., 2022). Recent network analysis researchers have focused on the single network structure of emotional distress in breast cancer survivors, such as the fear of cancer recurrence (Luo et al., 2022; Richter et al., 2022). However, a network analysis of associations between different psychological structures, such as emotional distress and cognitive factors, in breast cancer survivors is still lacking.

This study employed a network analysis approach to explore the relationship between psychological distress (depression, anxiety, and stress) and cognitive failure in breast cancer survivors. The central and bridge symptoms in the network were identified to provide a novel viewpoint for psychological interventions in breast cancer survivors.

## 2 Materials and methods

### 2.1 Participants

Breast cancer survivors receiving treatment or rehabilitation care were recruited between April and October 2019 from two hospitals in Weifang, Shandong Province. After being assessed by physicians, participants who met the criteria were introduced to the researchers. Trained researchers explained the survey to the participants. The survey commenced with the agreement of the participants. The inclusion criteria were as follows: (a) being 18 years of age or older; (b) having a physician-confirmed diagnosis of breast cancer; (c) possessing normal or corrected vision and the ability to comprehend and communicate; (d) being able to complete the questionnaire independently or with the help of caregivers; and (e) having awareness of their illness and participating voluntarily. The exclusion criteria were as follows: (a) having a history of other serious physical problems; (b) having mental illnesses or a family history of mental illnesses; and (c) having a history of drug/substance abuse.

### 2.2 Measure

#### 2.2.1 Psychological distress

The Depression Anxiety Stress Scales (DASS-21), developed by Lovidbond and validated in a Chinese setting (Chan et al., 2012), was used to measure psychological distress. The scale comprises 3 subscales and 21 items to measure depression, anxiety, and stress. The sum of the seven items in each subscale multiplied by two is the score of the subscale, which ranges from 0 to 42. Higher scores indicate more severe depression, anxiety, and stress. In this study, Cronbach's alpha of the DASS-21 was 0.925.

#### 2.2.2 Cognitive failure

The Cognitive Failure Questionnaire (CFQ) contains 25 items on the daily frequency of errors that patients may encounter. The five dimensions of the CFQ are distractibility, memory, interpersonal blunders, motor coordination, and memory for names. The scoring options indicate the frequency of these errors, ranging from never (0) to often (4). The total score of the CFQ ranges from 0 to 100. Higher scores indicate more serious self-reported cognitive failure (Zhou et al., 2016). Cronbach's alpha for cognitive failure was 0.917.

### 2.3 Data analysis

The data analyses were performed using SPSS 25.0 (IBM) and R version 4.1.2 (R Foundation for Statistical Computing). The descriptive statistics of the data were conducted using SPSS 25.0, and the relationship between the variables was examined using R version 4.1.2. In this study, each subscale score of the CFQ for cognitive failure and each subscale score of the DASS for psychological distress were continuous variables that were set as nodes in the network structure.

#### 2.3.1 Network estimation

Network estimations were conducted and plotted using the R package ggm, which refers to Gaussian Graphical Models (GGMs) with mixed graphs (Foygel and Drton, 2010), and R package qgraph (Epskamp et al., 2012), respectively. Edges were estimated using the EBICglasso package to establish the network structure (Janková and van de Geer, 2018). The edge represents the partial correlation between two nodes while controlling for the influence of all other nodes in the network. The combination of the GLasso algorithm with the EBIC criterion results in high sensitivity and specificity for both sparse graphs and large sample sizes (i.e., *n* > 250) (Epskamp and Fried, 2018). The thickness of the edges reflects the strength of the correlation between nodes, with thicker edges indicating higher partial correlation coefficients. This approach allows researchers to effectively identify and visualize the relationships between variables in the network.

#### 2.3.2 Network centrality

The node expected influence is considered the node centrality indicator, given the presence of positive and negative edges in the network. The expected influence of a node is calculated by summing the values of all the edges attached to the node. A higher expected influence indicates that the variable represented by that node played a more crucial role in the network. The package qgraph was used to estimate the expected influence of the node (Epskamp et al., 2012). Additionally, the predictability of all the nodes was plotted (Haslbeck and Fried, 2017). The level of predictability was represented by the circle surrounding each node, indicating how much the node can be explained by the variations in the connected nodes.

#### 2.3.3 Bridge centrality

Bridge centrality is an indicator for identifying the bridge node that connects two distinct communities in a network. Bridge nodes play a crucial role in facilitating interconnection between two communities. The bridge expected influence is one of the statistics used to identify the centrality of bridge nodes in a network. The bridge expected influence is calculated by summing the edge weights of one node connected to all nodes of other communities, utilizing the bridge function in R package networktools (Jones et al., 2021). A higher bridge expected influence of the node implies its significant role in connecting the current community with other communities (Jones et al., 2021). This research used *a priori*-defined community of variables, including the cognitive failure community and the psychological distress community.

#### 2.3.4 Network accuracy and stability

The bootnet package was employed to examine the stability and accuracy of the network (Epskamp et al., 2018). A case-dropping subset bootstrap was used to evaluate the correlation stability (CS) coefficient with 1,000 iterations. The measure represented the proportion of cases that could be dropped with a correlation of 0.70 in the node order for node centrality between the original and new networks, estimated after dropping the cases. This method was conducted to test the stability of the expected influence and bridge expected influence to confirm the robustness of the centrality indices. CS coefficient values above 0.25 and 0.5 were considered acceptable and robust (Epskamp et al., 2018). Additionally, all bootstrapped edge weights with 95% confidence intervals were estimated to verify the accuracy of the edge weights. Finally, the bootstrapped difference test for edge weights and node centralities was calculated.

## 3 Results

### 3.1 Sample characteristics

A total of 409 breast cancer survivors were included in the study. The median age of the survivors was 44.7 years. The majority of the survivors were married (*n* = 376, 91.93%) and had one child (*n* = 226, 55.26%). Most survivors had a senior high school education (*n* = 173, 42.30%) and were unemployed (*n* = 208, 50.85%), with a monthly income of *CNY* ¥1,000–3,000 (*n* = 178, 43.52%). In total, 270 (66.51%) survivors were diagnosed for < 1 year, 87 (21.27%) for 1–3 years, and 50 (12.22%) for more than 3 years. The demographic and clinical information of the participants is shown in Table 1.

**Table 1:** Demographic and clinical information of the sample (*n* = 409).

**Variables**	***n (%)***
**Age**	
< 30 years	17 (4.16%)
30–39 years	44 (10.76%)
40–49 years	157 (38.39%)
50–59 years	134 (32.76%)
60–69 years	46 (11.25%)
>69 years	11 (2.69%)
**Marital status**	
Married	376 (91.93%)
Divorced/Separated/Widowed	33 (8.07%)
**Child**	
0	26 (6.36%)
1	226 (55.26%)
≥2	157 (38.39%)
**Education**	
Primary school and below	55 (13.45%)
Junior High School	140 (34.23%)
Senior High school	173 (42.3%)
College and above	41 (10.02%)
**Occupational status**	
Employed	92 (22.49%)
Retired	109 (26.66%)
Unemployed	208 (50.85%)
**Family monthly income (** ***CNY*** **)**	
< 1,000	52 (12.71%)
1,000-3,000	178 (43.52%)
3,000- 5,000	106 (25.92%)
>5,000	73 (17.82%)
**Surgery**	
No	51 (12.47%)
Yes	358 (87.53%)
**Stage of cancer**	
Unknown	135 (33.01%)
I	115 (28.12%)
II	81 (19.8%)
III	21 (5.13%)
IV	57 (13.94%)
**Time since diagnosis**	
< 1 year	272 (66.51%)
1–3 years	87 (21.27%)
>3 years	50 (12.22%)
**Treatment**	
Chemotherapy	227 (55.5%)
Radiotherapy	60 (16.9%)
Endocrine/targeted therapy	120 (29.30%)
Others	32 (7.80%)
**Recurrence or metastasis**	
No	362 (88.51%)
Yes	47 (11.49%)

### 3.2 Descriptive statistics and correlation of variables

In total, 82 (20.05%) participants reported moderate or higher depression, 159 (38.88%) reported moderate or higher anxiety, and 60 (14.67%) reported moderate or higher stress. Psychological distress, including depression, anxiety, and stress, was positively associated with cognitive failure in all dimensions. The mean scores and correlation coefficients for all variables are shown in Table 2.

**Table 2:** Descriptive statistics and correlation for the variables (*n* = 409).

**Variables**	**M (SD)**	**1**	**2**	**3**	**4**	**5**	**6**	**7**	**8**
1 CFQ distractibility	25.92 (5.75)	1							
2 CFQ memory	14.62 (3.84)	0.77^**^	1						
3 CFQ interpersonal blunders	10.28 (2.76)	0.69^**^	0.62^**^	1					
4 CFQ motor coordination	6.50 (2.05)	0.69^**^	0.70^**^	0.54^**^	1				
5 CFQ memory for names	5.51 (1.67)	0.64^**^	0.56^**^	0.50^**^	0.48^**^	1			
6 DASS depression	7.16 (7.28)	0.40^**^	0.36^**^	0.37^**^	0.40^**^	0.29^**^	1		
7 DASS anxiety	8.32 (6.5)	0.37^**^	0.33^**^	0.30^**^	0.38^**^	0.24^**^	0.66^**^	1	
8 DASS stress	11.45 (7.38)	0.37^**^	0.30^**^	0.38^**^	0.28^**^	0.22^**^	0.69^**^	0.70^**^	1

M, mean score; SD, standard deviation; DASS, Depression Anxiety Stress Scales; CFQ, Cognitive Failure Questionnaire. ^**^P < 0.01.

### 3.3 Network structure

**Figure 1:**
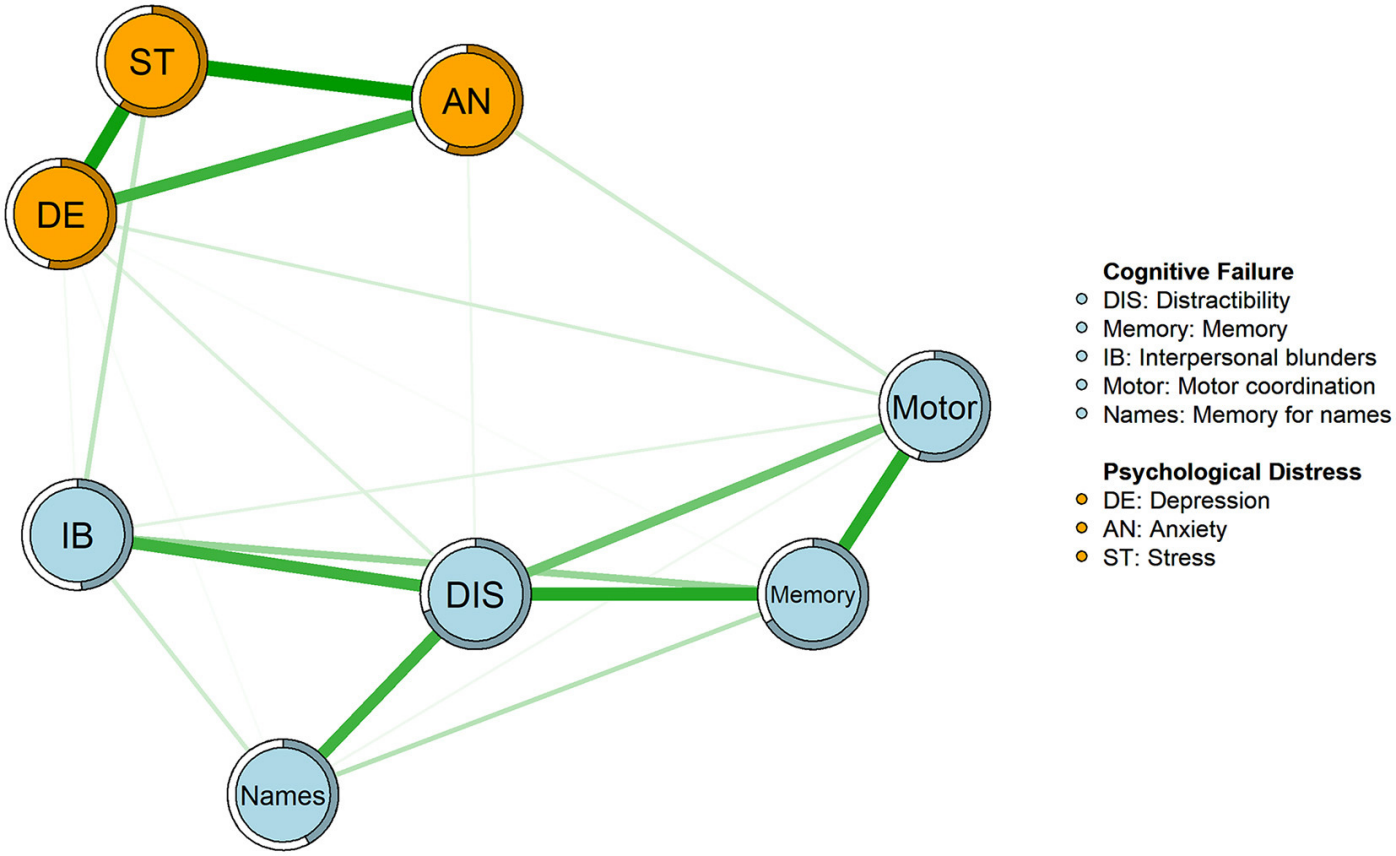
Network structure of psychological distress and cognitive failure. In the diagram, light blue nodes represent factors of cognitive failure, and orange nodes represent factors of psychological distress. The thickness of an edge indicates the degree of correlation between two nodes. Green edges represent positive correlations. Rings with a similar color around the nodes depict their predictability.

The network structure of psychological distress and cognitive failure in breast cancer survivors is shown in Figure 1. In the network, 21 out of 28 edges were estimated to be above zero. The edges of anxiety–stress (edge weight = 0.40), depression–stress (edge weight = 0.38), and distractibility–memory (edge weight = 0.35) were the strongest edges in the network. The edges of interpersonal blunders–stress (edge weight = 0.10), motor–anxiety (edge weight = 0.07), and motor–depression (edge weight = 0.07) connected the two communities with relatively lower edge weights. The bootstrapped 95% confidence intervals denoted the reliable accuracy of the edge weights (Supplementary Figure S1). In addition, the bootstrapped difference tests of edge weight in the network are shown in Supplementary Figure S2.

**Figure 2:**
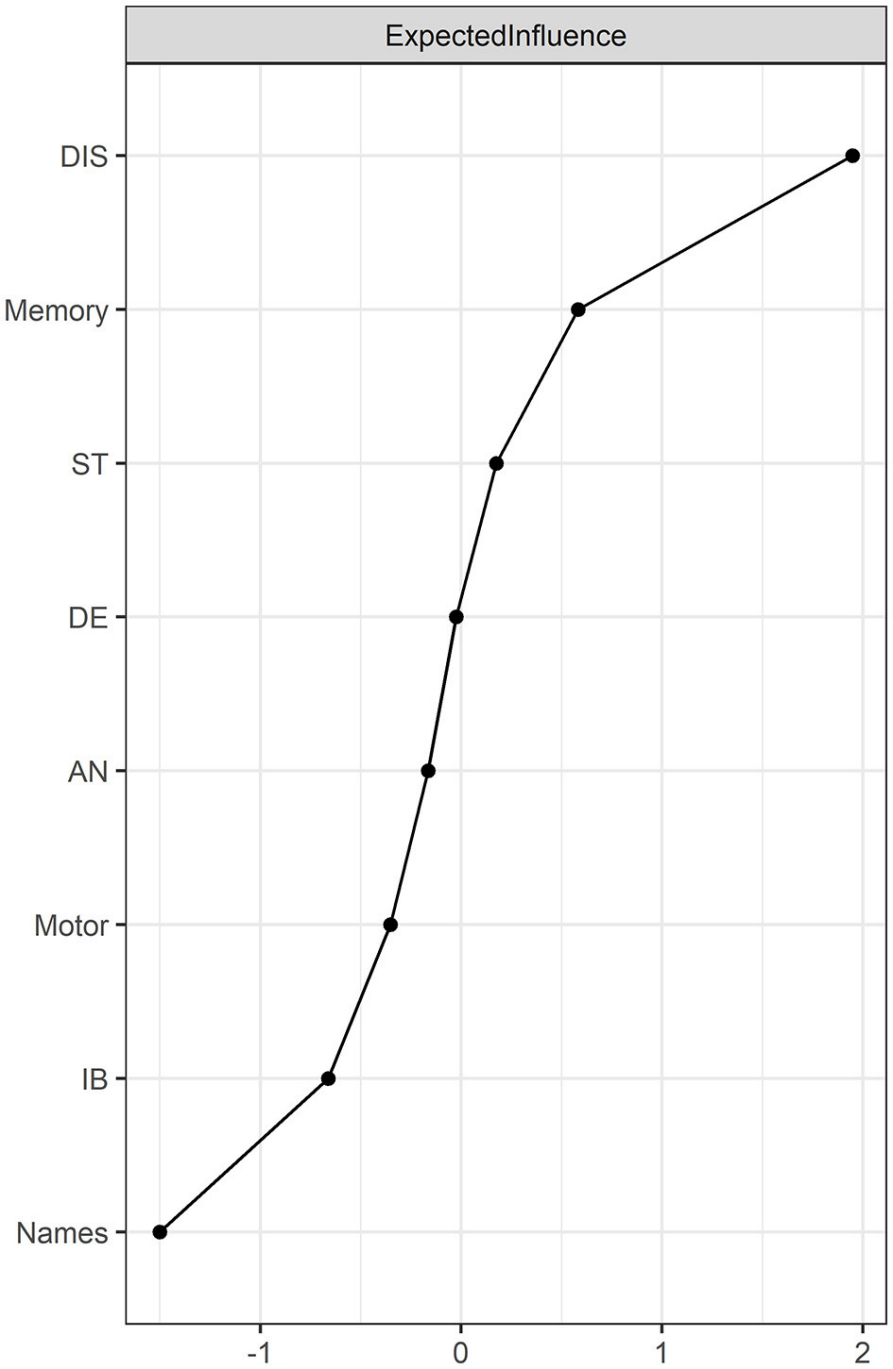
Node expected influence in the network (z-score). Node expected influence represents the centrality of the nodes. A higher centrality indicates the node has stronger connections to other nodes. DIS, Distractibility; Memory, Memory; IB, Interpersonal blunders; Motor, Motor coordination; Names, Memory for names; DE, Depression; AN, Anxiety; ST, Stress.

The node expected influence is shown in order of value in Figure 2. The node distractibility was the most central node, with the highest expected influence, followed by memory, stress, and depression, indicating that these nodes were the most connected to other nodes in the present network. The CS coefficient of node-expected influences was 0.75, which is considered highly stable (Supplementary Figure S3). The predictability of the nodes is depicted as the ring around the nodes in the network in Figure 1. The node predictability ranged from 42% to 70%, and the average predictability was 56%, indicating that, on average, 56% of the node variation could be interpreted by the neighboring nodes in the network. Moreover, the bootstrapped difference tests of expected influence revealed that the expected influence of distractibility was significantly higher (Supplementary Figure S4).

**Figure 3:**
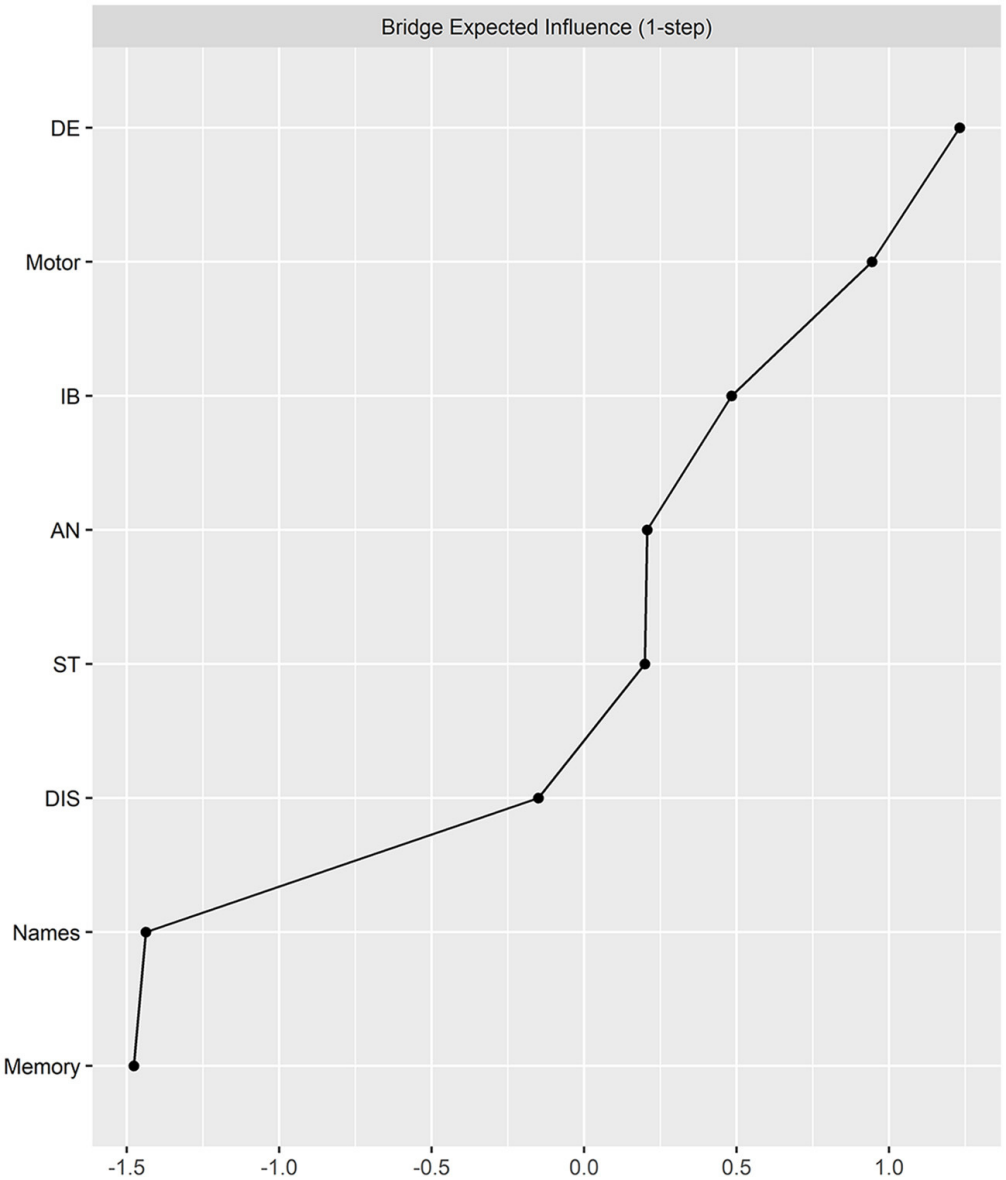
Bridge expected influence in the network (z-score). Bridge expected influence represents the bridge centrality of the nodes connected to different communities. A higher bridge expected influence indicates that the node in one community has stronger connections to other communities. DIS, Distractibility; Memory, Memory; IB, Interpersonal blunders; Motor, Motor coordination; Names, Memory for names; DE, Depression; AN, Anxiety; ST, Stress.

The bridge expected influence is shown in Figure 3. Depression and motor coordination were the strongest bridge nodes, with the highest bridge expected influences. This finding indicated that depression had the strongest connection with the cognitive failure community, while motor coordination had the strongest connection with the psychological distress community. The CS coefficient of the bridge expected influence was 0.21, which was slightly lower than the acceptable value (i.e., 0.25) (Supplementary Figure S5). Moreover, the bootstrapped difference test of bridge expected influence is shown in Supplementary Figure S6.

## 4 Discussion

To the best of our knowledge, this study was the first to explore the relationship between psychological distress and cognitive failure using the network analytical approach among Chinese breast cancer survivors. In the regularized partial correlation network, we identified the network structure and examined the node centrality, bridge centrality, and edge weights. Estimating the network between cognitive failure and psychological distress may offer novel insights into understanding mental health in breast cancer survivors as well as provide suggestions for improving the quality of survival for breast cancer survivors.

The strongest edges were between anxiety–stress, depression–stress, and distractibility–memory, suggesting that these nodes with stronger links tend to appear simultaneously. Consistent with previous findings, anxiety and depression in breast cancer survivors were significantly interconnected in the network (Hernandez et al., 2021; Yang et al., 2022). The correlations between anxiety and stress and between depression and stress were more significant than the correlation between anxiety and depression, which was slightly different from the findings of a previous study (van den Bergh et al., 2021). Breast cancer survivors face significant challenges beyond the stress induced by the physical symptoms of the disease; they also experience considerable psychological distress related to changes in body image and sexual role identity (Chang et al., 2019a,b, 2022; Todorov et al., 2019). Thus, the relationship between stress and other types of psychological distress is stronger in breast cancer survivors. The edge between distractibility and memory had the strongest association among the internal factors of cognitive failure. Notably, long-term attention and memory loss were the most common and debilitating cognitive symptoms that most breast cancer survivors experienced after chemotherapy (Bradley-Garcia et al., 2022). Women diagnosed with breast cancer and given adjuvant therapy were primarily affected by impairment in their concentration and memory, and a few reported an objective deterioration over time (Jenkins et al., 2006; Kim et al., 2023). Our results implied that the breast cancer survivors experienced memory decline, which was likely to be accompanied by attention loss in their daily lives.

Despite the relatively lower edge weights observed at the edges connecting the two communities, their existence still had significant implications. The bridges connecting the communities of psychological distress and cognitive failure were interpersonal blunders–stress, motor coordination–anxiety, and motor coordination–depression. Interpersonal blunders and motor coordination reflect an individual's errors of action (Pollina et al., 1992; Veal et al., 2023). The high levels of psychological distress experienced by breast cancer survivors occupy cognitive resources and induce errors in both physical and interpersonal activities. Such impaired performances in daily life may contribute to the negative emotions experienced by breast cancer survivors. These findings were similar to previous research, indicating that breast cancer survivors with higher levels of depression experience significantly more cognitive failures (Jenkins et al., 2006; West et al., 2022). Individuals who experience cognitive failure also more frequently report higher levels of anxiety and depression, both in cross-sectional and longitudinal study designs (Power, 1988; Merckelbach et al., 1996), and the influence may be especially severe if they are exposed to stressful situations, suggesting that individuals with cognitive impairment may have difficulties using effective coping strategies to deal with psychological distress (Broadbent et al., 1982; Boscher et al., 2020).

In breast cancer survivors, distractibility and memory were the most central nodes in the mapped network, followed closely by depression and stress, indicating that these components were especially notable in their connections with other variables in the network. Distractibility refers to the interference from internalized concentration or a lack of attention, and memory refers to the failure to retrieve information from memory in daily life (Pollina et al., 1992). Their high centrality suggested that targeting distractibility and memory could benefit other nodes in the network. Researchers have pointed out that attention and memory deficits are the basis of cognitive failures in daily life (Pollina et al., 1992). Memory plays a more salient and applicable role in daily mental activities. Better memory helps increase the perception of control. Impaired memory leads to important information being forgotten more frequently, which may have negative consequences (Knight et al., 2020). Individuals lacking the cognitive control capacity to regulate their working memory may show impairment in processing negative events, leading to increased psychological distress (Bridger et al., 2011). Similar explanations could be relevant to the role of distractibility. Breast cancer survivors experience an increased likelihood of errors in attention and memory. Cognitive failure could affect an individual's confidence and increase their psychological distress. When individuals experience cognitive failure in everyday life, they may attribute the failure to their lack of competence, make negative self-evaluations, form automatic cognitive biases, and further report higher psychological distress (Pfeifer et al., 2009). Previous research has revealed that memory is primarily associated with depression and anxiety and could be considered an important target in the cognitive treatment of patients with major depressive disorder (Knight et al., 2020). The findings of the present study indicate that distractibility and memory should be considered crucial targets for cognitive treatment to alleviate psychological distress in breast cancer survivors.

In the symptoms network, the symptoms in one disorder with high bridge centrality could increase the risk of co-occurrence with other disorders; therefore, interventions targeting the bridge symptoms could prevent comorbidity (Jones et al., 2021). Bridge nodes in the network may explain the interconnection between different communities and could be considered intervention targets to prevent activation across communities (Kaiser et al., 2021). The most significant bridge nodes in the network were depression in the psychological distress community and motor coordination in the cognitive failure community. Depression was associated with subjective cognitive failure (Zullo et al., 2021). Deficits in cognitive control capacity could increase the risk of depressive symptoms such as rumination due to low mood, poor performance, and negative evaluations of events (Bridger et al., 2011). Breast cancer survivors experience higher rates of depression due to the emotional impact of the diagnosis and treatment, changes in body image, and the adverse side effects of adjuvant therapy. The characteristics of depression include the perception of losing control and worthlessness (Zahn et al., 2015). Depression could be considered a vital consequence of cognitive failure in future studies or clinical practice. Motor coordination represents errors in daily behaviors due to poor motor coordination (Zhou et al., 2016). Errors in daily action can be more detrimental to an individual's self-esteem than errors in attention or memory, leading to self-doubt and psychological distress. An error of action activates a significant negative response, followed by increased concerns and self-control, as well as increased attention to performance, leading to further lapses. This vicious cycle could explain the maintenance of psychological distress (Farrin et al., 2003). Motor coordination should be considered an important pathway through which cognitive failure affects psychological distress. Therefore, interventions aimed at improving motor coordination might decrease the effect of cognitive failure on psychological distress. In summary, these findings indicate the bridge nodes in the joint relationship network between cognitive failure and psychological distress in breast cancer survivors.

Our findings provide important insights into the relationships between psychological characteristics in breast cancer survivors. Comorbidities of anxiety, depression, and stress were common in breast cancer survivors and were closely associated with cognitive performance (Li et al., 2021). Therefore, clinical caregivers should focus on the mental health and cognitive performance of breast cancer survivors to identify those who need specialized care. Breast cancer survivors with more frequent distractibility and memory errors were more likely to exhibit psychological distress. Motor coordination may play a vital role in cognitive failure, which in turn can lead to psychological distress. It is imperative to focus on cognitive changes throughout the treatment and recovery of breast cancer survivors.

There are some limitations to this study. First, the cross-sectional design prevented us from capturing longitudinal changes in the relationships over time. To address this limitation, future research could employ longitudinal data to elucidate the dynamics among variables. Second, the correlation stability coefficient of the bridge expected influence was 0.21; a value above 0.25 is considered acceptable (Epskamp et al., 2018). Although the coefficient in the present study is relatively low, it still provides significant insights. However, the results should be interpreted with caution. Finally, the generalizability of the findings may be constrained due to the limited focus on breast cancer survivors. Further studies could consider larger and more diverse samples to enhance the generalizability and explore potential variations in the network structure of the research variables across different populations.

## 5 Conclusion

Utilizing network analysis, our research elucidated the fine-grained associations between specific components of cognitive failure and psychological distress among breast cancer survivors. Distractibility and memory were the most central nodes, which played important roles in activating and maintaining the relationship network of cognitive failure and psychological distress. Motor coordination may be a key pathway by which cognitive failure impacts psychological distress. Our findings suggest that interventions targeting cognitive failure may be particularly efficient in preventing and decreasing psychological distress. Adjusting and controlling for central nodes, such as distractibility and memory, could decrease the connections between cognitive failure and psychological distress and therefore might be more effective in improving psychological distress. Furthermore, interventions targeting the bridge nodes, such as motor coordination, may reduce the extent to which cognitive failure triggers psychological distress.

## Data availability statement

The raw data supporting the conclusions of this article will be made available by the authors, without undue reservation.

## Ethics statement

The studies involving humans were approved by the Research Ethics Committee of Weifang Medical University. The studies were conducted in accordance with the local legislation and institutional requirements. The participants provided their written informed consent to participate in this study.

## Author contributions

BH: Conceptualization, Data curation, Formal analysis, Investigation, Methodology, Visualization, Writing – original draft. JY: Formal analysis, Writing – review & editing. RX: Conceptualization, Formal analysis, Investigation, Methodology, Validation, Visualization, Writing – original draft. MW: Conceptualization, Data curation, Funding acquisition, Writing – review & editing. JLiu: Conceptualization, Resources, Supervision, Writing – review & editing. LJ: Conceptualization, Methodology, Supervision, Writing – review & editing. JD: Conceptualization, Software, Validation, Writing – review & editing. XL: Conceptualization, Software, Writing – review & editing. HF: Formal analysis, Writing – review & editing. JLi: Resources, Writing – review & editing. CZ: Resources, Writing – review & editing. XS: Resources, Writing – review & editing. HD: Investigation, Writing – review & editing. YM: Investigation, Writing – review & editing. ST: Investigation, Writing – review & editing. NJ: Conceptualization, Funding acquisition, Software, Writing – review & editing. GL: Conceptualization, Funding acquisition, Project administration, Supervision, Writing – review & editing.

## References

[B1] Borsboom D. (2008). Psychometric perspectives on diagnostic systems. J. Clin. Psychol. 64, 1089–1108. 10.1002/jclp.20503 18683856

[B2] Boscher C. Joly F. Clarisse B. Humbert X. Grellard J.-M. Binarelli G.. (2020). Perceived cognitive impairment in breast cancer survivors and its relationships with psychological factors. Cancers 12:3000. 10.3390/cancers12103000 33081111 PMC7602817

[B3] Bradley-Garcia M. Winocur G. Sekeres M. J. (2022). Episodic Memory and recollection network disruptions following chemotherapy treatment in breast cancer survivors: a review of neuroimaging findings. Cancers 14:4752. 10.3390/cancers14194752 36230678 PMC9563268

[B4] Bridger R. S. Brasher K. Dew A. Sparshott K. (2011). Cumulative psychological strain and future strain in Naval personnel: is executive function the elephant in the room? Ergonomics 54, 597–608. 10.1080/00140139.2011.583361 21770748

[B5] Broadbent D. E. Cooper P. F. FitzGerald P. Parkes K. R. (1982). The cognitive failures questionnaire (CFQ) and its correlates. Br. J. Clin. Psychol. 21, 1–16. 10.1111/j.2044-8260.1982.tb01421.x 7126941

[B6] Brück E. Larsson J. W. Lasselin J. Bottai M. Hirvikoski T. Sundman E.. (2019). Lack of clinically relevant correlation between subjective and objective cognitive function in ICU survivors: a prospective 12-month follow-up study. Crit. Care 23:253. 10.1186/s13054-019-2527-1 31300016 PMC6625117

[B7] Carrigan N. Barkus E. (2016). A systematic review of cognitive failures in daily life: healthy populations. Neurosci. Biobehav. Rev. 63, 29–42. 10.1016/j.neubiorev.2016.01.010 26835660

[B8] Chan R. C. K. Xu T. Huang J. Wang Y. Zhao Q. Shum D. H. K.. (2012). Extending the utility of the Depression Anxiety Stress scale by examining its psychometric properties in Chinese settings. Psychiatry Res. 200, 879–883. 10.1016/j.psychres.2012.06.041 22921506

[B9] Chang Y.C. Hu W.Y. Chang Y.M. Chiu S.C. (2019b). Changes in sexual life experienced by women in Taiwan after receiving treatment for breast cancer. Int. J. Qual. Stud. Health Well-being 14:1654343. 10.1080/17482631.2019.1654343 31526246 PMC6758685

[B10] Chang Y. C. Chang S. R. Chiu S. C. (2019a). Sexual problems of patients with breast cancer after treatment: a systematic review. Cancer Nurs. 42, 418–425. 10.1097/NCC.0000000000000592 29621025

[B11] Chang Y. C. Lin G. M. Yeh T. L. Chang Y. M. Yang C. H. Lo C.. (2022). Impact of mindfulness-based stress reduction on female sexual function and mental health in patients with breast cancer. Support. Care Cancer 30, 4315–4325. 10.1007/s00520-021-06540-y 35092484 PMC8799961

[B12] Epskamp S. Borsboom D. Fried E.I. (2018). Estimating psychological networks and their accuracy: a tutorial paper. Behav. Res. Methods 50, 195–212. 10.3758/s13428-017-0862-1 28342071 PMC5809547

[B13] Epskamp S. Cramer A. O. J. Waldorp L. J. Schmittmann V. D. Borsboom D. (2012). qgraph: network visualizations of relationships in psychometric data. J. Stat. Softw. 48, 1–18. 10.18637/jss.v048.i04

[B14] Epskamp S. Fried E. I. (2018). A tutorial on regularized partial correlation networks. Psychol. Methods 23, 617–634. 10.1037/met0000167 29595293

[B15] Farrin L. Hull L. Unwin C. Wykes T. David A. (2003). Effects of depressed mood on objective and subjective measures of attention. J. Neuropsychiatry Clin. Neurosci. 15, 98–104. 10.1176/jnp.15.1.98 12556579

[B16] Fisher J. E. Zhou J. Liu A. G. Fullerton C. S. Ursano R. J. Cozza S. J. (2020). Effect of comorbid anxiety and depression in complicated grief on perceived cognitive failures. Depress. Anxiety 37, 54–62. 10.1002/da.22943 31916661

[B17] Foygel R. Drton M. (2010). Extended Bayesian information criteria for Gaussian graphical models. Adv. Neural Inf. Process. Syst. 23.

[B18] Franconeri S. L. Alvarez G. A. Cavanagh P. (2013). Flexible cognitive resources: competitive content maps for attention and memory. Trends Cogn. Sci. 17, 134–141. 10.1016/j.tics.2013.01.010 23428935 PMC5047276

[B19] Fried E. I. van Borkulo C. D. Cramer A. O. J. Boschloo L. Schoevers R. A. Borsboom D. (2017). Mental disorders as networks of problems: a review of recent insights. Soc. Psychiatry Psychiatr. Epidemiol. 52, 1–10. 10.1007/s00127-016-1319-z 27921134 PMC5226976

[B20] Hashemi S. M. Rafiemanesh H. Aghamohammadi T. Badakhsh M. Amirshahi M. Sari M.. (2020). Prevalence of anxiety among breast cancer patients: a systematic review and meta-analysis. Breast Cancer 27, 166–178. 10.1007/s12282-019-01031-9 31828585

[B21] Haslbeck J. M. B. Fried E. I. (2017). How predictable are symptoms in psychopathological networks? A reanalysis of 18 published datasets. Psychol. Med. 47, 2767–2776. 10.1017/S0033291717001258 28625186

[B22] Hernandez M. O. S. Carrasco M. A. Holgado-Tello F. P. (2021). Anxiety and depression symptoms in Spanish children and adolescents: an exploration of comorbidity from the network perspective. Child Psychiatry Hum. Dev. 1–14. 34797464 10.1007/s10578-021-01286-4 PMC10140092

[B23] Janková J. van de Geer S. (2018). “Inference in high-dimensional graphical models,” in Handbook of graphical models (CRC Press), 325–350. 10.1201/9780429463976-14

[B24] Jenkins V. Shilling V. Deutsch G. Bloomfield D. Morris R. Allan S.. (2006). A 3-year prospective study of the effects of adjuvant treatments on cognition in women with early stage breast cancer. Br. J. Cancer 94, 828–834. 10.1038/sj.bjc.6603029 16523200 PMC3216421

[B25] Jones P. J. Ma R. F. McNally R. J. (2021). Bridge centrality: a network approach to understanding comorbidity. Multivariate Behav. Res. 56, 353–367. 10.1080/00273171.2019.1614898 31179765

[B26] Kaiser T. Herzog P. Voderholzer U. Brakemeier E.-L. (2021). Unraveling the comorbidity of depression and anxiety in a large inpatient sample: network analysis to examine bridge symptoms. Depress. Anxiety 38, 307–317. 10.1002/da.23136 33465284

[B27] Kim H. J. Kim J. E. E. Jung S. O. Lee D. Abraham I. (2023). Neuropsychological effects of chemotherapy: systematic review of longitudinal studies on objective cognitive impairment in breast cancer patients. Cancer Nurs. 46, E159–e168. 10.1097/NCC.0000000000001079 35324504

[B28] Knight M. J. Lyrtzis E. Baune B. T. (2020). The association of cognitive deficits with mental and physical quality of life in major depressive disorder. Compr. Psychiatry 97:152147. 10.1016/j.comppsych.2019.152147 31838296

[B29] Li J. Gao W. Yang Q. Cao F. (2021). Perceived stress, anxiety, and depression in treatment-naive women with breast cancer: a case-control study. Psychooncology 30, 231–239. 10.1002/pon.5555 32969126

[B30] Luo X. Li W. A. Chen Y. Sun H. W. Humphris G. Liu T.. (2022). Fear of recurrence in Chinese cancer patients: prevalence, correlates, and network analysis. Front. Psychiat. 13:803543. 10.3389/fpsyt.2022.803543 35197876 PMC8859333

[B31] Merckelbach H. Muris P. Nijman H. de Jong P. J. (1996). Self-reported cognitive failures and neurotic symptomatology. Pers. Individ. Dif. 20, 715–724. 10.1016/0191-8869(96)00024-4

[B32] Otto A. K. Soriano E. C. Siegel S. D. LoSavio S. T. Laurenceau J. P. (2018). Assessing the relationship between fear of cancer recurrence and health care utilization in early-stage breast cancer survivors. J. Cancer Surviv. 12, 775–785. 10.1007/s11764-018-0714-8 30341560

[B33] Payne T. W. Schnapp M. A. (2014). The relationship between negative affect and reported cognitive failures. Depress. Res. Treat. 2014:396195. 10.1155/2014/396195 24669318 PMC3942281

[B34] Pfeifer S. van Os J. Hanssen M. Delespaul P. Krabbendam L. (2009). Subjective experience of cognitive failures as possible risk factor for negative symptoms of psychosis in the general population. Schizophr. Bull. 35, 766–774. 10.1093/schbul/sbn004 18296431 PMC2696365

[B35] Pilevarzadeh M. Amirshahi M. Afsargharehbagh R. Rafiemanesh H. Hashemi S. M. Balouchi A. (2019). Global prevalence of depression among breast cancer patients: a systematic review and meta-analysis. Breast Cancer Res. Treat. 176, 519–533. 10.1007/s10549-019-05271-3 31087199

[B36] Pollina L. K. Greene A. L. Tunick R. H. Puckett J. M. (1992). Dimensions of everyday memory in young adulthood. Br. J. Psychol. 83, 305–321. 10.1111/j.2044-8295.1992.tb02443.x 1393363

[B37] Power M. J. (1988). Cognitive failures, dysfunctional attitudes, and symptomatology: a longitudinal study. Cogn. Emot. 2, 133–143. 10.1080/02699938808408070

[B38] Pullens M. J. De Vries J. Roukema J. A. (2010). Subjective cognitive dysfunction in breast cancer patients: a systematic review. Psychooncology 19, 1127–1138. 10.1002/pon.1673 20020424

[B39] Richter D. Clever K. Mehnert-Theuerkauf A. Schönfelder A. (2022). Fear of recurrence in young adult cancer patients-a network analysis. Cancers 14:2092. 10.3390/cancers14092092 35565220 PMC9105535

[B40] Rogers M. L. Hom M. A. Joiner T. E. (2019). Differentiating acute suicidal affective disturbance (ASAD) from anxiety and depression Symptoms: a network analysis. J. Affect. Disord. 250, 333–340. 10.1016/j.jad.2019.03.005 30875677

[B41] Sun K. Lei L. Zheng R. Zhang S. Zeng H. Wang S.. (2023). Trends in incidence rates, mortality rates, and age-period-cohort effects of female breast cancer - China, 2003-2017. China CDC Wkly 5, 340–346. 10.46234/ccdcw2023.065 37193084 PMC10182910

[B42] Sung H. Ferlay J. Siegel R. L. Laversanne M. Soerjomataram I. Jemal A.. (2021). Global cancer statistics 2020: GLOBOCAN estimates of incidence and mortality worldwide for 36 cancers in 185 countries. CA Cancer J. Clin. 71, 209–249. 10.3322/caac.21660 33538338

[B43] Todorov N. Sherman K. A. Kilby C. J. Breast Canc Network A. (2019). Self-compassion and hope in the context of body image disturbance and distress in breast cancer survivors. Psychooncology. 28, 2025–2032. 10.1002/pon.5187 31369188

[B44] van den Bergh N. Marchetti I. Koster E. H. W. (2021). Bridges over troubled waters: mapping the interplay between anxiety, depression and stress through network analysis of the DASS-21. Cognit. Ther. Res. 45, 46–60. 10.1007/s10608-020-10153-w

[B45] Veal B.M. Scott S.B. Jim H.S.L. Small B.J. (2023). Subjective cognition and memory lapses in the daily lives of breast cancer survivors: examining associations with objective cognitive performance, fatigue, and depressed mood. Psychooncology 32, 1298–1305. 10.1002/pon.6185 37381150 PMC10859854

[B46] Wang Y. H. Li J. Q. Shi J. F. Que J. Y. Liu J. J. Lappin J. M.. (2020). Depression and anxiety in relation to cancer incidence and mortality: a systematic review and meta-analysis of cohort studies. Mol. Psychiatry 25, 1487–1499. 10.1038/s41380-019-0595-x 31745237

[B47] West T. Cavallero C. Ceccherini R. Foladore S. Generali D. Versace F.. (2022). Impact of psychosocial, behavioral and lifestyle factors on subjective cognitive complaints and perceived quality of life in a large cohort of Italian breast cancer patients. Front. Psychol. 13:1015573. 10.3389/fpsyg.2022.1015573 36438336 PMC9683534

[B48] Wirkner J. Weymar M. Loew A. Hamm C. Struck A.-M. Kirschbaum C.. (2017). Cognitive functioning and emotion processing in breast cancer survivors and controls: an ERP pilot study. Psychophysiology 54, 1209–1222. 10.1111/psyp.12874 28432781

[B49] Xu C. Ganesan K. Liu X. Ye Q. Cheung Y. Liu D.. (2022). Prognostic value of negative emotions on the incidence of breast cancer: a systematic review and meta-analysis of 129,621 patients with breast cancer. Cancers 14:475. 10.3390/cancers14030475 35158744 PMC8833353

[B50] Yang Y. Sun H. W. Luo X. Li W. A. Yang F. Xu W. J.. (2022). Network connectivity between fear of cancer recurrence, anxiety, and depression in breast cancer patients. J. Affect. Disord. 309, 358–367. 10.1016/j.jad.2022.04.119 35472477

[B51] Zahn R. Lythe K. E. Gethin J. A. Green S. Deakin J. F. W. Young A. H.. (2015). The role of self-blame and worthlessness in the psychopathology of major depressive disorder. J. Affect. Disord. 186, 337–341. 10.1016/j.jad.2015.08.001 26277271 PMC4573463

[B52] Zhou Y. Chen J. Z. Liu Y. Wang P. Zhu L. J. Yan T. H. (2016). Validity and reliability of the cognitive failures questionnaire in Chinese college students. Chinese J. Clin. Psychol. 24, 438–443. 10.16128/j.cnki.1005-3611.2016.03.013

[B53] Zinchenko A. Kanske P. Obermeier C. Schroger E. Kotz S. A. (2015). Emotion and goal-directed behavior: ERP evidence on cognitive and emotional conflict. Soc. Cogn. Affect. Neurosci. 10, 1577–1587. 10.1093/scan/nsv050 25925271 PMC4631156

[B54] Zullo L. Clark C. Gholam M. Castelao E. von Gunten A. Preisig M.. (2021). Factors associated with subjective cognitive decline in dementia-free older adults-A population-based study. Int. J. Geriatr. Psychiatry 36, 1188–1196. 10.1002/gps.5509 33555636

