# Supplementary material for: The relationship between psychological distress and cognitive failure among breast cancer survivors: a network analysis
Source: Front Psychol. 2024 Jul 10;15:1420125. doi: 10.3389/fpsyg.2024.1420125 (PMC11271155; doi:10.3389/fpsyg.2024.1420125)
Supplement: Supplementary file 1 [file Image_1.PDF]

Supplementary Material

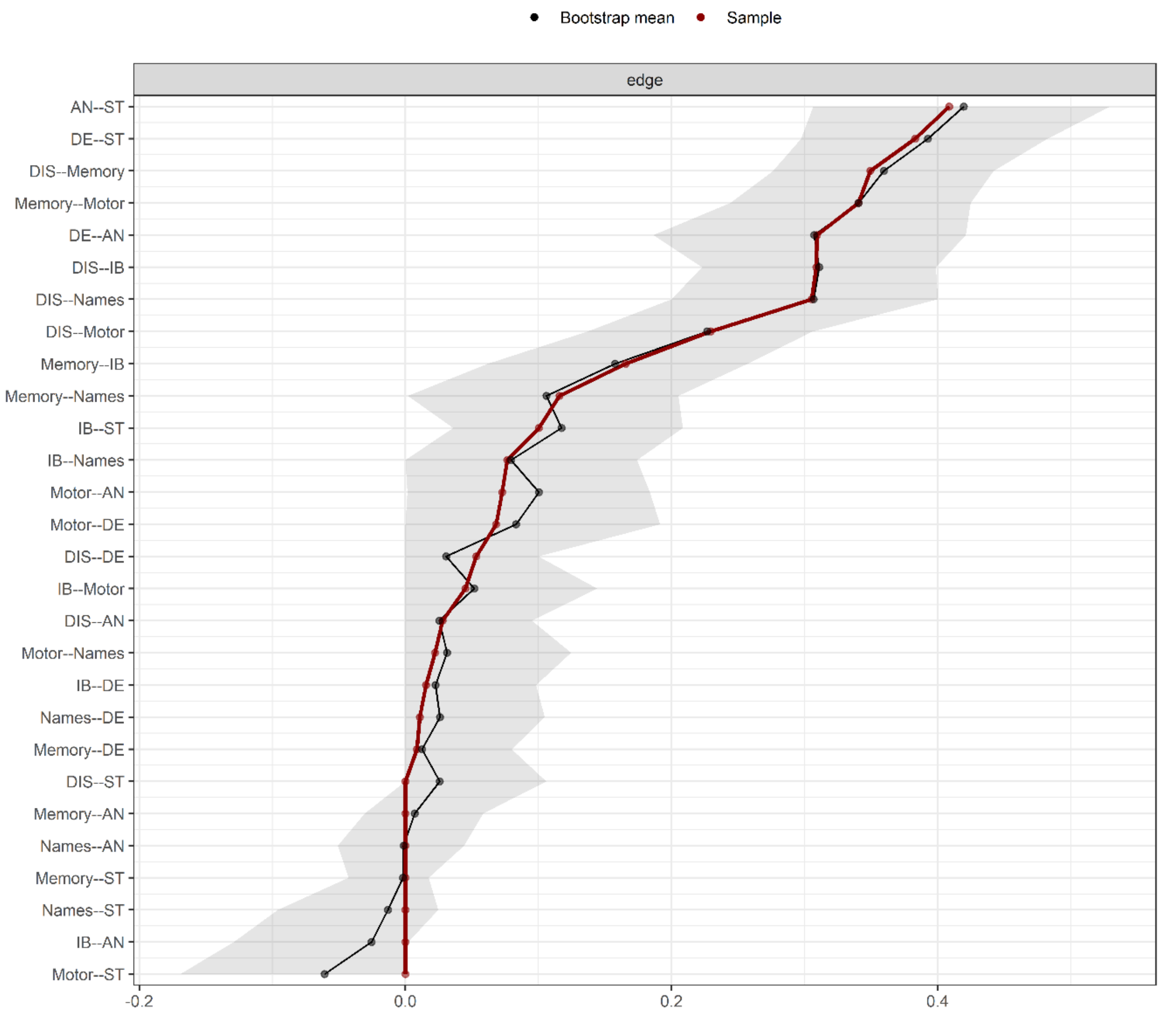

Supplementary Figure S1. Edge weight accuracy test of the network using the non-parametric bootstrapping method.

Note: The sample edge-weight is illustrated by red line and the bootstrap mean edge-weight is illustrated by black line. The grey area indicates the bootstrapped confidence intervals. Narrower confidence interval represents reliable accuracy. DIS=Distractibility; Memory=Memory; IB=Interpersonal blunders; Motor=Motor coordination; Names=Memory for names; DE=Depression; AN=Anxiety; ST=Stress.

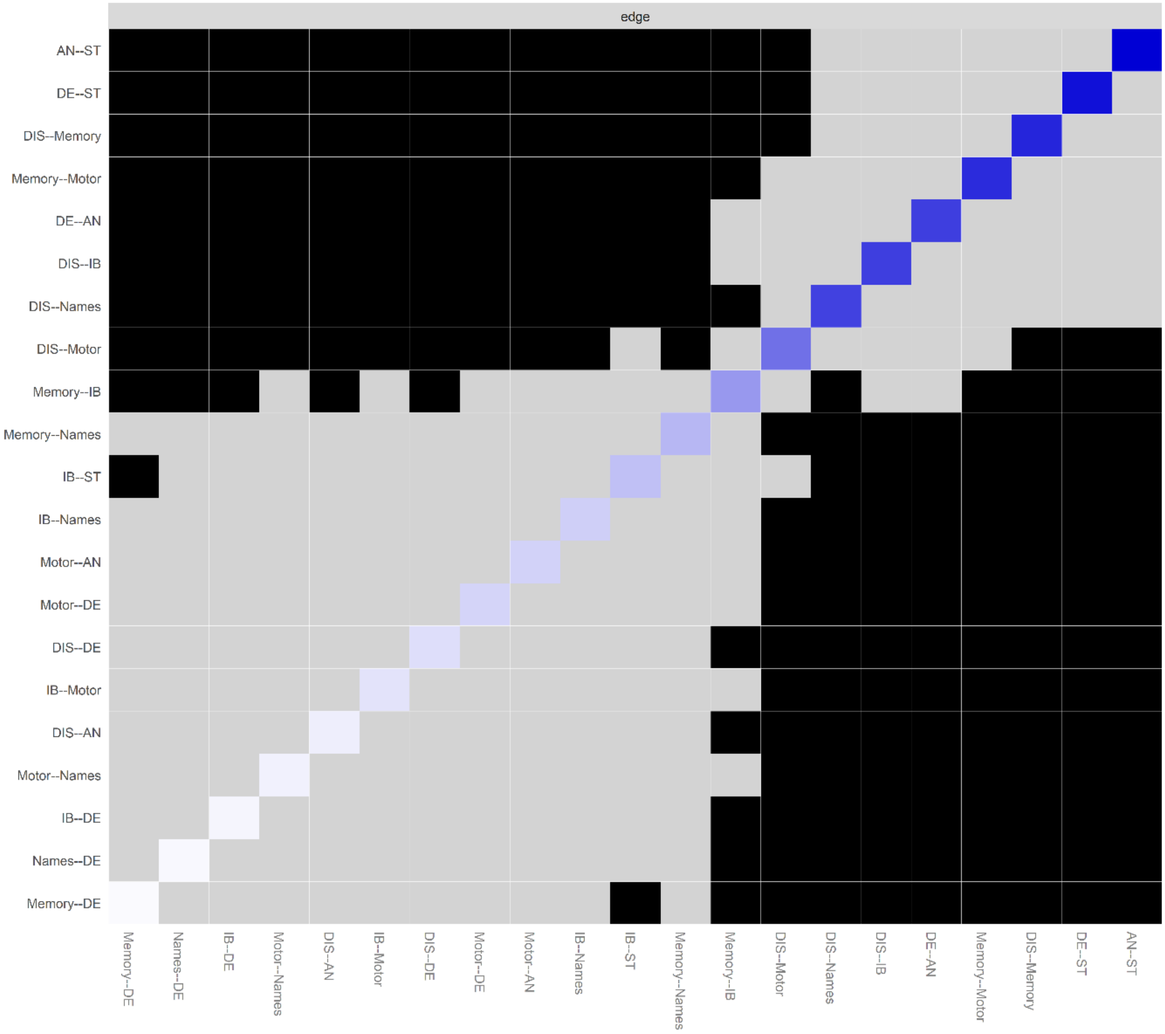

Supplementary Figure S2. Differences in edge weight using the non-parametric bootstrapping method

Note: The plot shows significant differences of edge weight between the edges. The black grid indicates that edge weight of the edge is significantly different from another edges, and the grey grid indicates no significant difference. DIS=Distractibility; Memory=Memory; IB=Interpersonal blunders; Motor=Motor coordination; Names=Memory for names; DE=Depression; AN=Anxiety; ST=Stress.

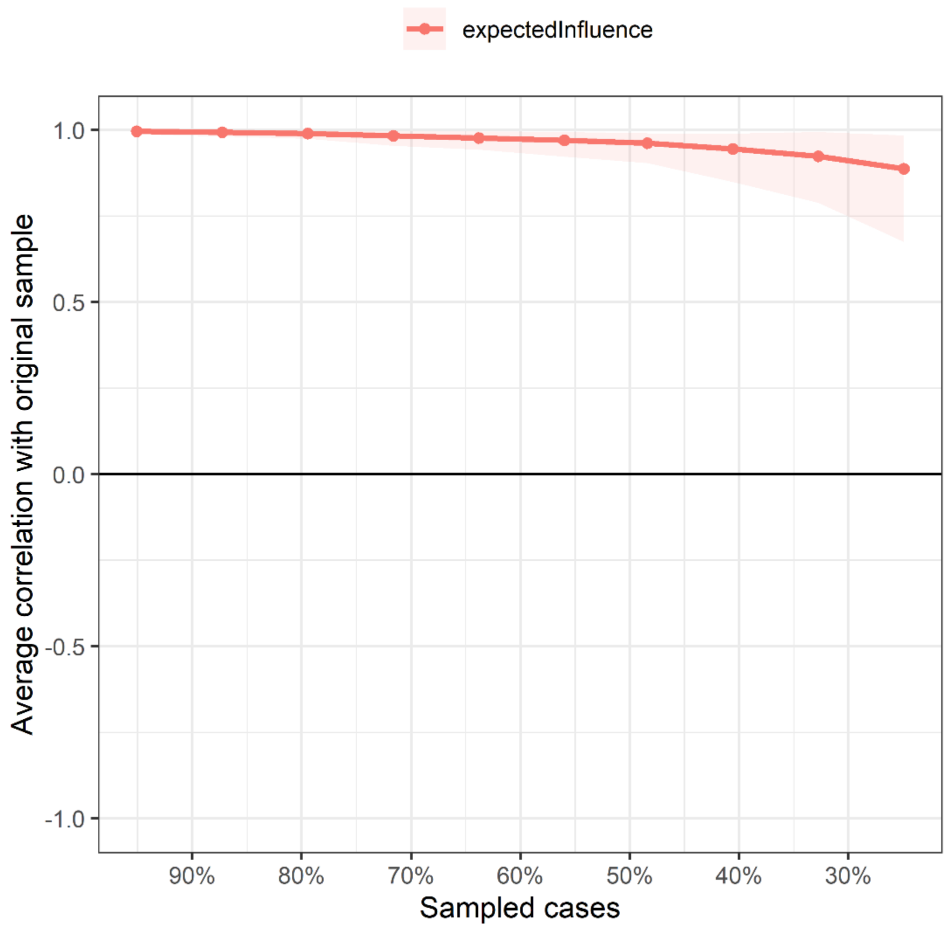

Supplementary Figure S3. Stability for node expected influences using the case-drop bootstrapping method.

Note: The red line indicates the averaged correlation between node expected influences of the original sample and subsample. The red area represents the range from 2.5th quantile to the 97.5th quantile.

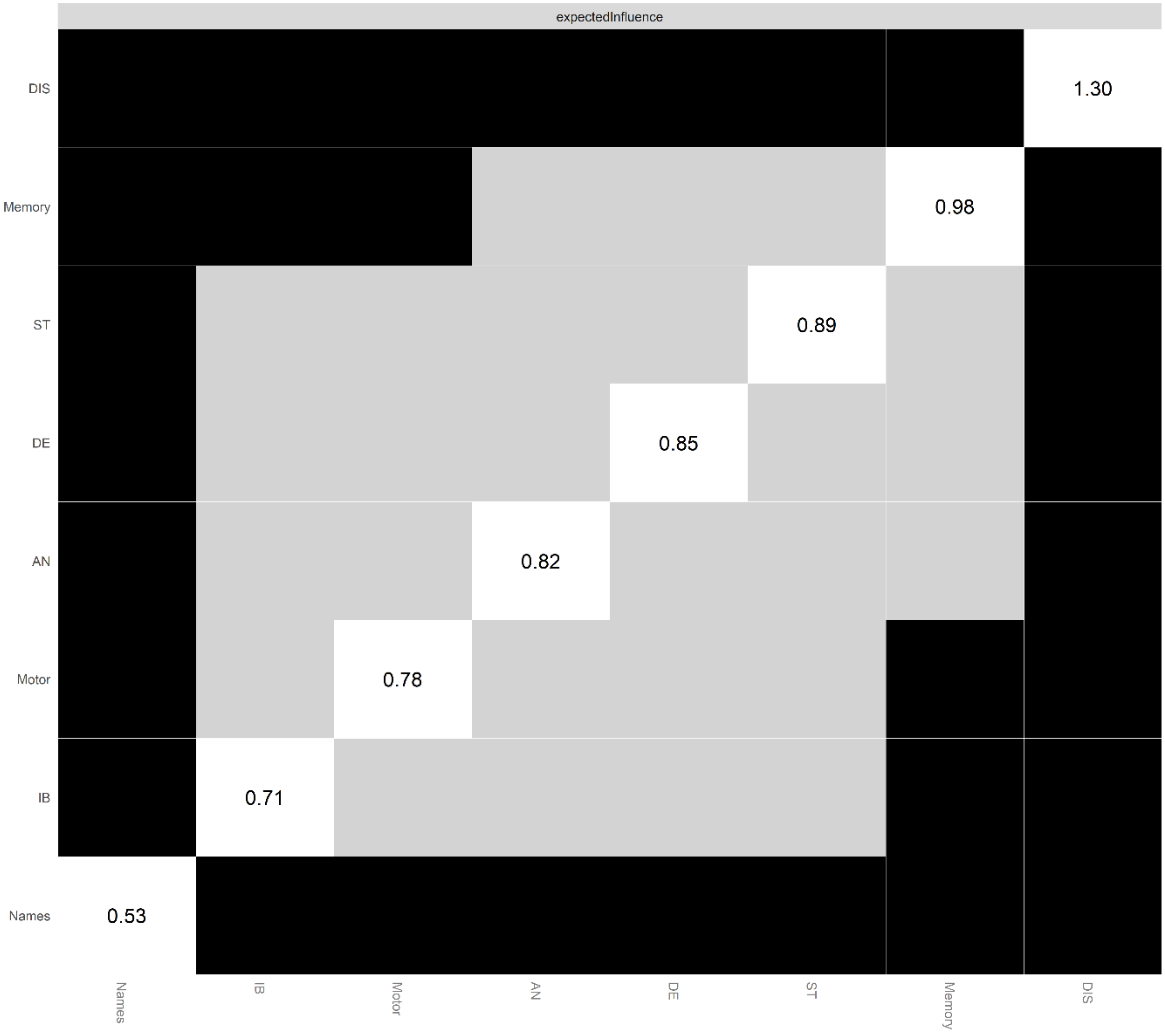

Supplementary Figure S4. Differences test results of node expected influences using the non-parametric bootstrapping method.

Note: The plot shows significant differences between the expected influence of the nodes. The black grid indicates that the expected influence of a node is significantly different from another nodes, and the grey grid indicates no significant difference. DIS=Distractibility; Memory=Memory; IB=Interpersonal blunders; Motor=Motor coordination; Names=Memory for names; DE=Depression; AN=Anxiety; ST=Stress.

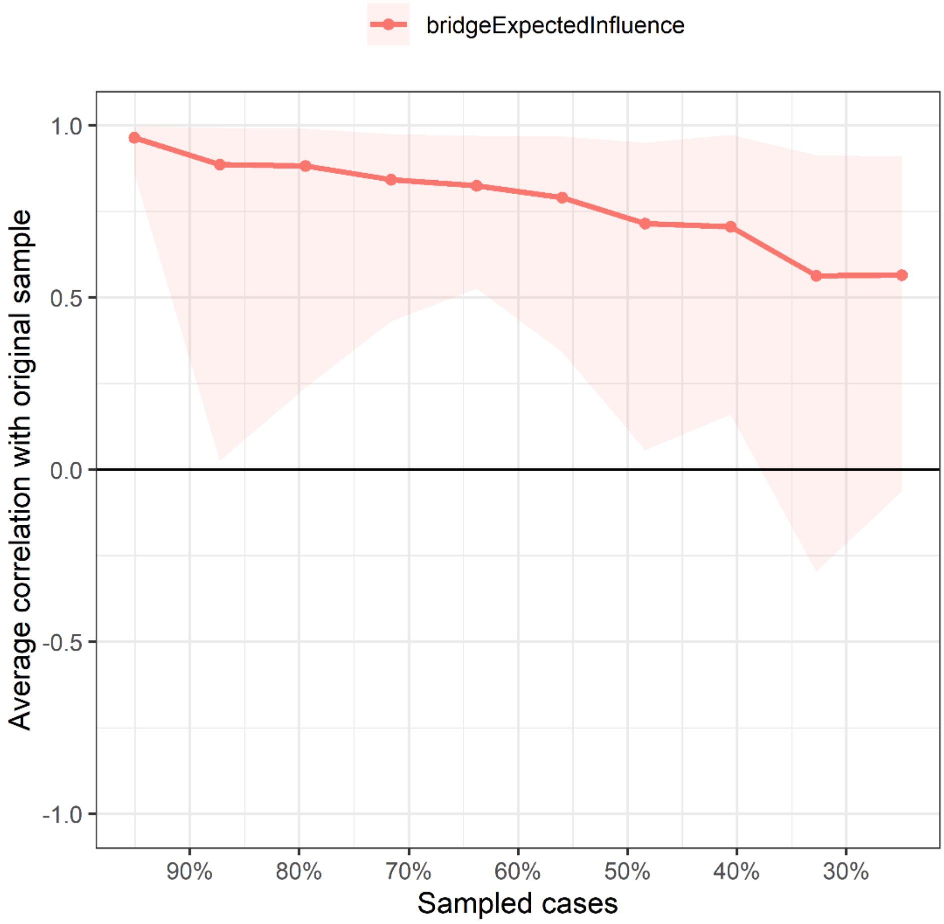

Supplementary Figure S5. Stability for bridge expected influences using the case-drop bootstrapping method.

Note: The red line indicates the averaged correlation between bridge expected influences of the original sample and subsample. The red area represents the range from 2.5th quantile to the 97.5th quantile.

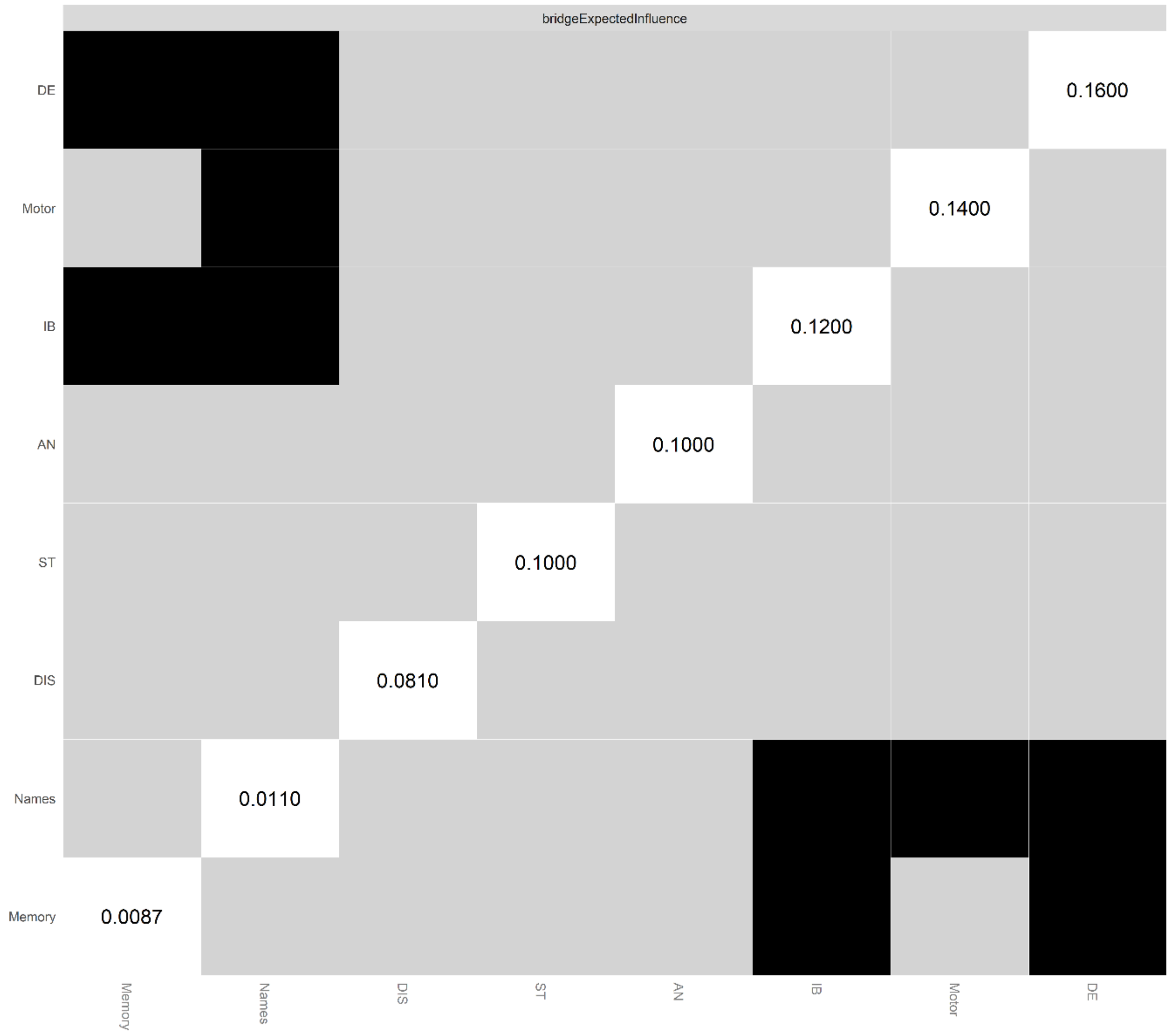

Supplementary Figure S6. Differences test results of bridge expected influences using the non-parametric bootstrapping method.

Note: The plot shows significant differences between the bridge expected influence of the nodes. The black square indicates that the bridge expected influence of a node is significantly different from another nodes, and the grey grid indicates no significant difference. DIS=Distractibility; Memory=Memory; IB=Interpersonal blunders; Motor=Motor coordination; Names=Memory for names; DE=Depression; AN=Anxiety; ST=Stress.
